# Brain metabolic stress and neuroinflammation at the basis of cognitive impairment in Alzheimer’s disease

**DOI:** 10.3389/fnagi.2015.00094

**Published:** 2015-05-19

**Authors:** Fernanda G. De Felice, Mychael V. Lourenco

**Affiliations:** Institute of Medical Biochemistry Leopoldo de Meis, Federal University of Rio de JaneiroRio de Janeiro, RJ, Brazil

**Keywords:** Alzheimer’s disease, amyloid-β oligomers, endoplasmic reticulum stress, inflammation, metabolic stress

## Abstract

Brain metabolic dysfunction is known to influence brain activity in several neurological disorders, including Alzheimer’s disease (AD). In fact, deregulation of neuronal metabolism has been postulated to play a key role leading to the clinical outcomes observed in AD. Besides deficits in glucose utilization in AD patients, recent evidence has implicated neuroinflammation and endoplasmic reticulum (ER) stress as components of a novel form of brain metabolic stress that develop in AD and other neurological disorders. Here we review findings supporting this novel paradigm and further discuss how these mechanisms seem to participate in synapse and cognitive impairments that are germane to AD. These deleterious processes resemble pathways that act in peripheral tissues leading to insulin resistance and glucose intolerance, in an intriguing molecular connection linking AD to diabetes. The discovery of detailed mechanisms leading to neuronal metabolic stress may be a key step that will allow the understanding how cognitive impairment develops in AD, thereby offering new avenues for effective disease prevention and therapeutic targeting.

## Introduction

Incidence of Alzheimer’s disease (AD) will greatly increase as world population ages (Prince et al., 2013) and changes in lifestyle observed in recent decades seem to be major contributors to such increased prevalence (Mattson, 2012; De Felice, 2013). Likewise, common diseases of modern adulthood, including obesity and diabetes mellitus, have been often regarded as AD risk factors (De Felice, 2013). Pioneering epidemiological studies connecting AD to diabetes initiated in the 1990s (Ott et al., 1996, 1999; Kalmijin et al., 1997) and were followed by several reports providing both clinical and experimental evidence into how these two disorders may course together (de la Monte, 2009; Matsuzaki et al., 2010; Crane et al., 2013; De Felice, 2013; De Felice et al., 2014).

Metabolic derangements, including inflammation, insulin resistance and endoplasmic reticulum (ER) stress, are known to underlie glucose intolerance and type 2 diabetes mellitus (T2DM) in peripheral tissues (Hotamisligil et al., 1995, 1996; Ozcan et al., 2004, 2006; Hotamisligil, 2006). A similar scenario has been recently described in the brains of patients that suffer from neurodegenerative disorders, such as AD. Neuropathology investigations have revealed that AD brains present several markers of insulin resistance, inflammation and ER stress (Hoozemans et al., 2005; Steen et al., 2005; Moloney et al., 2010; Bomfim et al., 2012; Talbot et al., 2012; O’Neill, 2013; De Felice et al., 2014). In the following sections, we review current evidence indicating that a newly defined form of metabolic stress leads the path to cognitive decline in AD. The understanding of molecular mechanisms driving AD pathogenesis may shed new light on novel targets for drug development and offer strategies for disease prevention.

## AD Pathogenesis

AD pathophysiology includes neuroinflammation, oxidative and ER stress, synapse loss and degeneration of specific neuronal populations (Selkoe, 2002; Ferreira and Klein, 2011; Mucke and Selkoe, 2012). Amyloid-β peptide (Aβ) is the main component of senile plaques that accumulate in AD brains (Masters et al., 1985), and substantial evidence indicates that Aβ is causally involved in AD (Mucke and Selkoe, 2012). Consolidated knowledge has established that soluble Aβ oligomers (AβOs; Lambert et al., 1998), and not necessarily the insoluble amyloid fibrils detected in senile plaques, promote direct damage to synapses, besides stimulating inflammatory response and cellular stress (Ferreira and Klein, 2011; Viola and Klein, 2015). These findings prompt AβOs, which are increased in AD brains (Gong et al., 2003; Xia et al., 2009), to be considered neurotoxins responsible for synapse and memory loss in AD early stages.

Very recent data has demonstrated that AβO actions stimulate pro-inflammatory mechanisms to impair neuronal insulin signaling and to trigger stress kinase activation, resulting in synapse and memory impairments in AD models (Bomfim et al., 2012; Lourenco et al., 2013; Ma et al., 2013). These events are quite similar to those acting in peripheral tissues to impair metabolism in diabetes and obesity (De Felice and Ferreira, 2014), in line with the idea that a form of metabolic stress develops in AD brains (Kapogiannis and Mattson, 2011; Yoon et al., 2012; De Felice and Ferreira, 2014). Such findings may impact translational research, as treating brain metabolic dysfunction might be a key strategy to fight neurological disorders.

## Brain Metabolic Stress Mechanisms in AD

In peripheral tissues, prolonged inflammatory cascades lead to the activation of multiple cellular stress mechanisms that ultimately impair cell function and body metabolism (Hotamisligil, 2006; Gregor and Hotamisligil, 2011). In AD, evidence arising from *in vitro*, *in vivo* and neuropathology studies supports that such events occur throughout disease development and are linked to AβO neurotoxicity. Oligomers promote neuronal stress by instigating abnormal elevations in levels of tumor necrosis factor α (TNF-α) and reactive oxygen species (ROS), as well as activation in JNK/PKR signaling and increased eIF2α phosphorylation (eIF2α-P) levels in AD models (De Felice et al., 2007, 2014; Ma et al., 2009, 2013; Bomfim et al., 2012; Lourenco et al., 2013). In this context, pro-inflammatory signals appear to be directly responsible for defective insulin signaling and stress-mediated synapse loss caused by AβOs in neurons (Bomfim et al., 2012; Lourenco et al., 2013). This has led to a concept in which AβOs build up in pre-AD brains to cause inflammation (e.g., gliosis and cytokine production) and neuronal metabolic stress, ultimately leading to synaptic dysfunction and behavioral alterations. We next detail some of the mechanisms recently implicated in AD pathogenesis.

### Unfolded Protein Response

Unfolded Protein Response (UPR) is defined as a collection of signaling pathways that respond to ER stress due to accumulation of misfolded proteins and/or impaired homeostasis. ER membrane sensors activate three signaling axes (ATF6α, IRE-1α/XBP-1s and PERK/eIF2α-P) to instigate transcriptional and translational alterations aimed at restoring cell homeostasis (Lai et al., 2007; Hetz et al., 2013). UPR signaling attenuates global translation and favors the synthesis of select transcription factors, such as ATF4, CHOP and Nrf2 (Buffington et al., 2014; Hetz and Mollereau, 2014). Under continued stress, however, these pathways may promote cell damage and death. This hormetic response pattern is thus critical to determine cell fate in such conditions (Mattson, 2008; Hetz, 2012).

Evidence for canonical UPR activation has been found in AD neurons (Hoozemans et al., 2005, 2009; Yoon et al., 2012) and in AD mouse models (Yoon et al., 2012; Ma et al., 2013). In accordance, AβOs trigger UPR in hippocampal neurons *in vitro* and *in vivo* (Chafekar et al., 2007; Casas-Tinto et al., 2011; Lourenco et al., 2013; Barbero-Camps et al., 2014), and experimental induction of ER stress leads to neuronal metabolic stress (Yoon et al., 2012), tau phosphorylation (Bose et al., 2011; van der Harg et al., 2014), stress kinase activation (Bose et al., 2011; Paquet et al., 2011) and cognitive impairment in mice (Lourenco et al., 2013). Further, alleviating ER stress with 4-phenylbutyrate, a chemical chaperone, promotes cognitive benefits in AD mouse models (Ricobaraza et al., 2009, 2010; Wiley et al., 2011; Lourenco et al., 2013).

**Figure 1:**
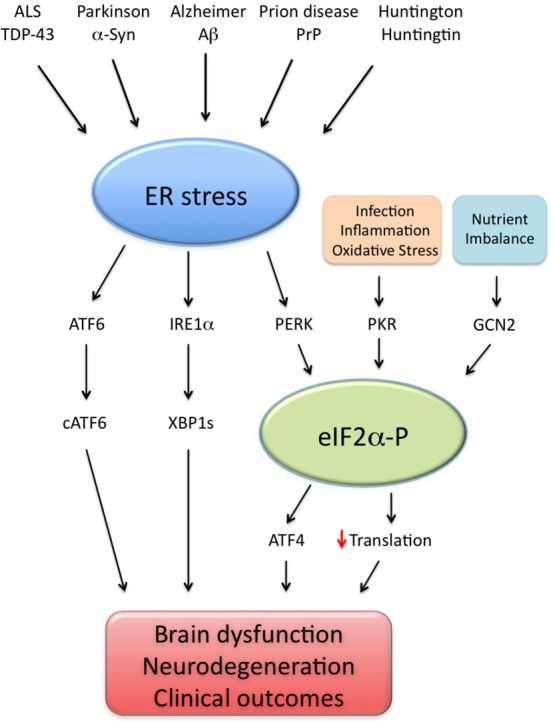
**Endoplasmic reticulum (ER) stress as a common denominator of neurodegenerative diseases**. Brain ER stress is caused by several disease-associated stressors, including amyloid-β (Aβ; Alzheimer disease), α-synuclein (α-syn; Parkinson disease), prion protein (PrP; prion-related diseases), mutated huntingtin (Huntington and poly-Q diseases) and TAR DNA-binding protein of 43 kDa (TDP-43; amyotrophic lateral sclerosis, ALS). In such disorders, abnormal ER stress leads to the activation of three signaling pathways (ATF6; IRE1α/XBP1 s and PERK/eIF2α-P) collectively termed unfolded protein response (UPR). In parallel, events of infection, inflammation, nutrient deprivation and oxidative stress activate additional eIF2α kinases (namely PKR and GCN2), leading to abnormally high eIF2α-P levels, increased ATF4 production and impaired translation. Excessive UPR activity, eIF2α-P signaling and their downstream effectors impair cell function and may result in brain dysfunction and neurodegeneration, possibly explaining the clinical outcomes observed in neurodegenerative conditions.

Substantial recent evidence has proposed that UPR activation is a common feature of different neurodegenerative diseases, as deleterious impacts of UPR branches were reported in AD (Lourenco et al., 2013; Ma et al., 2013; Barbero-Camps et al., 2014; van der Harg et al., 2014), Parkinson’s (Bellucci et al., 2011; Colla et al., 2012), Huntington (Lajole and Snapp, 2011), amyotrophic lateral sclerosis (ALS; Hetz et al., 2009; Kim et al., 2013) and prion diseases (Moreno et al., 2012). Correcting UPR activation further appears to be effective in preclinical models of prion infection (Moreno et al., 2012, 2013; Halliday et al., 2015) and ALS (Hetz et al., 2009; Kim et al., 2013), in addition to AD models (Ricobaraza et al., 2009, 2010; Lourenco et al., 2013; Ma et al., 2013). Therefore, it is likely that aberrant UPR signaling mediates brain dysfunction in a variety of neurological conditions (Figure 1).

### eIF2α-P and Translational Repression

Under cellular stress, translational repression can be mediated by increased eIF2α phosphorylation (eIF2α-P), a regulatory factor essential for translation initiation in eukaryotes (Raven and Koromilas, 2008). PERK-mediated eIF2α-P is the main UPR branch leading to general protein synthesis repression and facilitation of select mRNA translation (Buffington et al., 2014). Both PERK and eIF2α-P appear to be elevated in AD brains (Chang et al., 2002b; Yoon et al., 2012; Ma et al., 2013) and are induced by Aβ aggregates in neurons (Lee et al., 2010). Increased eIF2α-P has been further verified in other AD mouse models (Segev et al., 2012; Devi and Ohno, 2014).

Two other eIF2α kinases, namely the stress kinase PKR and the nutrient sensor GCN2, are enriched in the brain and have been reported to increase neuronal eIF2α-P (Costa-Mattioli et al., 2005; Lourenco et al., 2013; Roffé et al., 2013; Hetz and Mollereau, 2014), and thus emerge as candidates to explain increased eIF2α-P in AD.

Interestingly, deletion of either PERK or GCN2 in the brains of APP/PS1 mice decreases eIF2α-P levels, rescuing synapse plasticity and cognition (Ma et al., 2013). AβOs increase eIF2α-P through TNF-α-dependent PKR activation, thereby promoting synapse loss in hippocampal neurons and cognitive impairment in mice (Paquet et al., 2011; Lourenco et al., 2013). Providing clinical relevance to the findings observed in experimental models, PKR was found to be abnormally active in AD brains (Chang et al., 2002a; Paquet et al., 2011; Mouton-Liger et al., 2012b). Therefore, it is likely that PERK, GCN2, and PKR lead to increased eIF2α-P levels in AD.

Increased eIF2α-P levels also facilitate the translation of a small fraction of mRNAs (Buffington et al., 2014), among which is activating transcription factor 4 (ATF4), a protein linked to oxidative stress, enhanced γ-secretase activity and neuronal dysfunction when abnormally elevated (Mitsuda et al., 2007; Lange et al., 2008). ATF4 signaling further counteracts CREB1 pro-memory actions in mice (Costa-Mattioli et al., 2005; Rajasethupathy et al., 2012).

Recent findings demonstrated that ATF4 levels are increased in AD brains (Yoon et al., 2012; Baleriola et al., 2014) and in AD animal models (Ma et al., 2013; Devi and Ohno, 2014). Furthermore, soluble Aβ species appear to locally stimulate axonal ATF4 translation to propagate a neurodegenerative message in mice (Baleriola et al., 2014). Hence, eIF2α-P/ATF4 signaling has the potential to explain, at least in part, how disease progresses from defined brain regions in the beginning to a widespread forebrain dysfunction at later stages.

Translational repression instigated by eIF2α-P may be harmful to cognition, given that normal protein synthesis is required for memory (Flexner et al., 1964; Rossato et al., 2007). Accordingly, APP/PS1 mice present reduced brain protein synthesis in parallel to memory loss, and AβOs impair LTP-induced hippocampal protein synthesis (Ma et al., 2013). Nevertheless, the identity of memory-relevant translational products that are impacted in AD still remains to be determined.

### Stress Kinase Activation

Cellular stress is also known to activate a family of protein kinases that mediate adaptive responses (Calay and Hotamisligil, 2013). These proteins are termed stress-sensitive kinases (or simply stress kinases) and include JNK, p38 MAPK, PKR, PERK and IKK, among other serine/threonine kinases (Vallerie and Hotamisligil, 2010; Hetz and Mollereau, 2014). Active stress kinases phosphorylate several protein targets to restore homeostasis. Nevertheless, their excessive or prolonged actions may trigger cell injury and, later, programmed cell death (Mattson, 2008; Vallerie and Hotamisligil, 2010; Hetz, 2012; De Felice et al., 2014).

Neuropathology studies have demonstrated abnormal activation of neuronal stress-sensitive kinases in AD brains. Indeed, abnormal phosphorylation of p38 MAPK (Hensley et al., 1999), JNK (Ma et al., 2009; Bomfim et al., 2012; Yoon et al., 2012), PERK (Hoozemans et al., 2005, 2009), PKR (Chang et al., 2002a; Paquet et al., 2011) and IKK (Talbot et al., 2012) have been reported in AD brains and might be core mediators of neuronal dysfunction. Accordingly, AβOs have been described to activate neuronal JNK and PKR to impair insulin signaling and synapse function (Ma et al., 2009; Bomfim et al., 2012; Lourenco et al., 2013), and transgenic animal models of AD exhibit similar alterations in JNK and PKR activity (Ma et al., 2009; Bomfim et al., 2012; Lourenco et al., 2013). Consistently, blocking either PKR or the brain-enriched JNK3 rescue cognitive impairments in AD mouse models (Yoon et al., 2012; Lourenco et al., 2013), suggesting that stress kinase activation lies upstream of synapse and memory impairment in AD.

An attractive possibility is that PKR further drives the activation of other MAPKs, such as p38MAPK and JNK, thus exacerbating neuronal damage. Very recent findings suggest that the interaction between PKR and the RNA-binding protein TRBP is essential to promote eIF2α-P and JNK activation under obesity-induced metabolic stress (Nakamura et al., 2015). A similar scenario might also develop in AD even independently of TNF-α, given that oxidative stress has been reported to activate neuronal PKR (Mouton-Liger et al., 2012a). Moreover, AβOs could activate PKR in glial cells to instigate MAPK-dependent actions, exacerbating neuroinflammatory responses in AD brains. These notions still demand further investigation.

### Neuroinflammation

Elevated markers of inflammation are found in both AD animal models and human AD brains (Ferreira et al., 2014; Monson et al., 2014; Heneka et al., 2015). Consistently, evidence for gliosis and central infiltration of peripheral immune cells is often found in histopathological studies in AD mouse models (Yamanaka et al., 2012; Lourenco and Ledo, 2013; Yang et al., 2013; Baik et al., 2014; Ferreira et al., 2014; Monson et al., 2014).

Amyloid aggregates (ranging from oligomers to fibrils) induce a neuroinflammatory profile that may lead to synapse and neuronal damage (Combs, 2009; Pan et al., 2011; Lourenco et al., 2013; Medinas and Hetz, 2013; Parajuli et al., 2013; Heneka et al., 2015). Nevertheless, rather than deposited plaques, AβOs are thought to be core inducers of brain inflammation, given that they are potent microglial activators (Floden and Combs, 2006; Dhawan et al., 2012; Ledo et al., 2013) and diffuse throughout brain regions (Lambert et al., 1998; Forny-Germano et al., 2014; Viola and Klein, 2015). Accumulating evidence suggests that AβO-induced microglial activation releases TNF-α and other cytokines that, in turn, act on neurons to cause stress signaling and synapse injury (Floden and Combs, 2006; Sondag et al., 2009; Bomfim et al., 2012; Dhawan et al., 2012; Lourenco et al., 2013).

**Figure 2:**
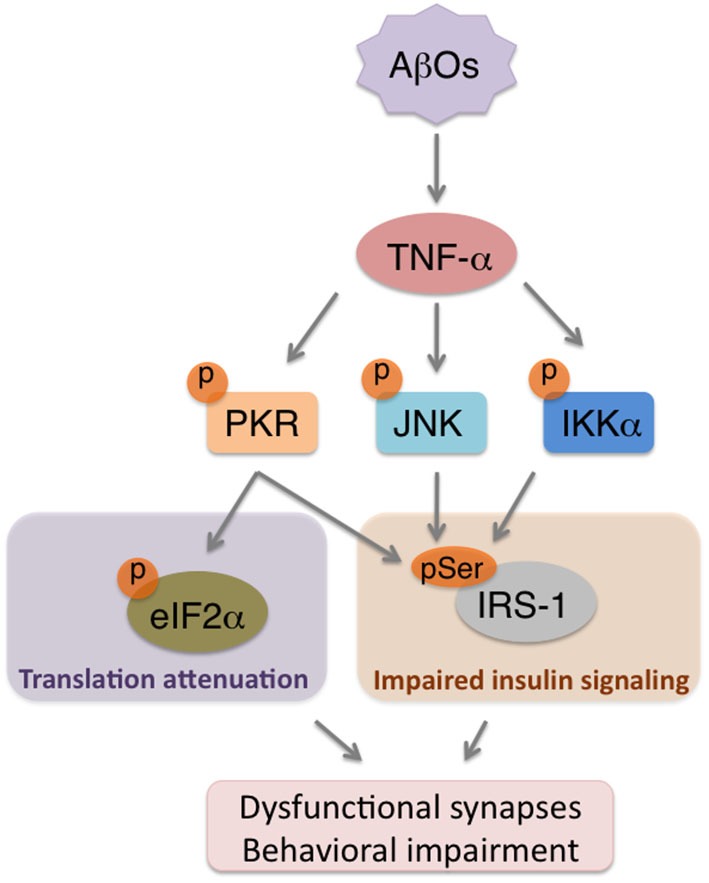
**AβOs trigger brain metabolic stress in Alzheimer’s Disease (AD)**. Accumulation of AβOs in pre-AD brains instigates an inflammatory response that involves increased TNF-α production. TNF-α, in turn, acts on neurons to promote the activity of stress kinases (e.g., PKR, JNK, IKKα), which will serine phosphorylate both eIF2α-P to attenuate translation, and IRS-1 to impair insulin signaling. The combination of repressed protein synthesis and defective insulin signaling are components of a novel form of neuronal metabolic stress that may contribute to synapse deregulation and cognitive impairment in AD.

Therefore, neuroinflammation is considered to take place over the degenerative course of AD and to be linked to cognitive dysfunction. In fact, our recent results showed that AβO-triggered elevations in TNF-α levels orchestrate neuronal stress mechanisms to impair brain insulin signaling (Bomfim et al., 2012), synapses and cognition in animal models of AD (Lourenco et al., 2013; Figure 2). This cascade is mediated by stress kinases, including JNK and PKR, in the brains affected by AβOs (Lourenco et al., 2013). Since evidence suggests that reducing neuroinflammation can counteract memory deficits in AD mouse models (Medeiros et al., 2007; McAlpine et al., 2009; Kiyota et al., 2010; Bachstetter et al., 2012), a more complete understanding of how brain inflammation develops may lead to effective targeting of aberrant mechanisms underlying cognitive symptoms in AD.

## Metabolic Stress and Cognitive Function in AD

Experimental evidence has gathered inflammation, defective insulin signaling and cell stress to AD-linked neurotoxicity and neurodegeneration in a revised concept of metabolic stress (Paquet et al., 2011; Mouton-Liger et al., 2012a; Yoon et al., 2012; Ledo et al., 2013; Lourenco et al., 2013; Ma et al., 2013; Baleriola et al., 2014; De Felice et al., 2014). Although the classical alterations in glucose metabolism germane to metabolic impairments are observed in AD brains (Hoyer et al., 1988; Kapogiannis and Mattson, 2011; Chen and Zhong, 2013), the modern notion of metabolic stress also includes disturbances in proteostasis and activation of signaling pathways that mediate cellular stress.

In this context, the progressive build-up of AβOs in AD brains might trigger the activation of immune mechanisms, including glial cell reactivity and cytokine release that, in turn, lead to neuronal metabolic stress. A point of convergence of multiple stress pathways is found on elevated eIF2α-P levels. Accordingly, PKR, ER stress, eIF2α-P and ATF4 have been described as negative modulators of memory (Costa-Mattioli et al., 2007; Zhu et al., 2011; Rajasethupathy et al., 2012; Lourenco et al., 2013; Stern et al., 2013; Di Prisco et al., 2014; Ounallah-Saad et al., 2014). By acting together, such pathways might disrupt brain homeostasis and contribute to the cognitive decline observed in AD.

The precise mechanisms linking metabolic stress to synapse defects are still not fully understood, but the findings that increased eIF2α-P levels lead to LTP impairments (Ma et al., 2013) and synapse loss (Lourenco et al., 2013) in mice have provided initial clues on this causal relationship. Consistently, restoring normal brain eIF2α-P levels was shown to abrogate deficient levels of synaptic proteins and cognition (Lourenco et al., 2013; Ma et al., 2013), indicating a tight connection between eIF2α-P and synapse/memory integrity.

It is noteworthy that activation of PKR/eIF2α-P signaling (O’Connor et al., 2008; Devi and Ohno, 2010; Mouton-Liger et al., 2012a), as well as high-fat diet-induced metabolic stress (Wang et al., 2013) was shown to promote amyloidogenesis in a feed-forward cycle that might exacerbate amyloid pathology. It is thus tempting to speculate that accumulating injuries throughout life, including infections, diabetes and obesity, could instigate a brain metabolic stress scenario that includes ER stress and neuroinflammation to facilitate Aβ accumulation and sporadic AD onset at later stages of life (Herrup, 2010; Mattson, 2012; De Felice, 2013).

An unresolved question relates to whether brain insulin resistance could itself trigger AD-related phenomena, even in the absence of inflammation. In this regard, early studies using neuronal insulin receptor knockout (NIRKO) mice found that deficient brain insulin signaling causes abnormal tau phosphorylation without spatial memory impairment (Schubert et al., 2004). Recently, NIRKO mice were shown to develop anxiety and depressive-like behavior linked to altered dopamine metabolism (Kleinridders et al., 2015), and deletion of a single gene copy that encodes an insulin receptor subunit in the brain impairs synaptic plasticity and cognition (Nisticò et al., 2012). Nonetheless, it remains to be determined whether such mice develop brain metabolic stress in the presence or absence of neurotoxic stimuli. Future investigation may dissect the molecular steps that are required for metabolic stress-induced synapse impairments in an AD context.

## Conclusions

Recent exciting evidence has connected AβO-induced neuronal stress to cognitive impairments in AD, in a mechanism that includes cytokine-induced activation of stress kinases and ultimately leads to neuronal and synapse dysfunction in AD experimental models (De Felice et al., 2007; Yoon et al., 2012; De Felice, 2013; Lourenco et al., 2013; Ma et al., 2013; Baleriola et al., 2014; De Felice and Ferreira, 2014; Ferreira et al., 2014). Hence, the combination of inflammation, neuronal insulin resistance, oxidative/ER stress and translational repression might generate a noxious scenario of brain metabolic stress to mediate and propagate synapse defects, resulting in cognitive deficits. In this context, ER stress and abnormal eIF2α-P levels emerged as key players in neuronal damage.

Sporadic AD is largely idiopathic, and it is noteworthy that Aβ-centric views of AD pathogenesis remain controversial (see Morris et al., 2014 for a critical review). Nonetheless, recent progress summarized here may have deep implications for disease prevention, as avoiding harmful events throughout life might reduce the risk of brain inflammation, metabolic stress and, consequently, of developing AD at later stages of life. Interrupting deleterious molecular pathways at prodromal stages will likely be the ideal strategy to delay AD progression. The identification of common AD drivers is imperative to establish effective therapeutics, and blocking neuronal metabolic stress at the earliest cognitive symptoms could offer a promising approach to minimize neuronal dysfunction and AD progression. Repurposing labeled anti-diabetic compounds could constitute an interesting option as they have been shown to attenuate AD-linked brain metabolic stress and memory dysfunction (Craft, 2012; De Felice et al., 2014). Future clinical trials may reveal whether these drugs, alone or in combination, are indeed effective in AD.

## Conflict of Interest Statement

The authors declare that the research was conducted in the absence of any commercial or financial relationships that could be construed as a potential conflict of interest.

## References

[B1] Bachstetter A. D. Norris C. M. Sompol P. Wilcock D. M. Goulding D. Neltner J. H.. (2012). Early stage drug treatment that normalizes proinflammatory cytokine production attenuates synaptic dysfunction in a mouse model that exhibits age-dependent progression of Alzheimer’s disease-related pathology. J. Neurosci. 32, 10201–10210. 10.1523/jneurosci.1496-12.2012 22836255 PMC3419360

[B2] Baik S. H. Cha M. Y. Hyun Y. M. Cho H. Hamza B. Kim D. K.. (2014). Migration of neutrophils targeting amyloid plaques in Alzheimer’s disease mouse model. Neurobiol. Aging 35, 1286–1292. 10.1016/j.neurobiolaging.2014.01.003 24485508 PMC4248665

[B3] Baleriola J. Walker C. A. Jean Y. Y. Crary J. F. Troy C. M. Nagy P. L.. (2014). Axonally synthesized ATF4 transmits a neurodegenerative signal across brain regions. Cell 158, 1159–1172. 10.1016/j.cell.2014.07.001 25171414 PMC4149755

[B4] Barbero-Camps E. Fernández A. Baulies A. Martinez L. Fernández-Checa J. C. Colell A. (2014). Endoplasmic reticulum stress mediates amyloid beta neurotoxicity via mitochondrial cholesterol trafficking. Am. J. Pathol. 184, 2066–2081. 10.1016/j.ajpath.2014.03.014 24815354 PMC4076561

[B5] Bellucci A. Navarria L. Zaltieri M. Falarti E. Bodei S. Sigala S.. (2011). Induction of the unfolded protein response by alpha-synuclein in experimental models of Parkinson’s disease. J. Neurochem. 116, 588–605. 10.1111/j.1471-4159.2010.07143.x 21166675

[B6] Bomfim T. R. Forny-Germano L. Sathler L. B. Brito-Moreira J. Houzel J. C. Decker H.. (2012). An anti-diabetes agent protects the mouse brain from defective insulin signaling caused by Alzheimer’s disease-associated Aβ oligomers. J. Clin. Invest. 122, 1339–1353. 10.1172/jci57256 22476196 PMC3314445

[B7] Bose A. Mouton-Liger F. Paquet C. Mazot P. Vigny M. Gray F.. (2011). Modulation of tau phosphorylation by the kinase PKR: implications in Alzheimer’s disease. Brain Pathol. 21, 189–200. 10.1111/j.1750-3639.2010.00437.x 21029237 PMC8094269

[B8] Buffington S. A. Huang W. Costa-Mattioli M. (2014). Translational control in synaptic plasticity and cognitive dysfunction. Annu. Rev. Neurosci. 37, 17–38. 10.1146/annurev-neuro-071013-014100 25032491 PMC4721605

[B9] Calay E. S. Hotamisligil G. S. (2013). Turning off the inflammatory, but not the metabolic, flames. Nat. Med. 19, 265–267. 10.1038/nm.3114 23467233

[B10] Casas-Tinto S. Zhang Y. Sanchez-Garcia J. Gomez-Velazquez M. Rincon-Limas D. E. Fernandez-Funez P. (2011). The ER stress factor XBP1s prevents amyloid- neurotoxicity. Hum. Mol. Genet. 20, 2144–2160. 10.1093/hmg/ddr100 21389082 PMC3090193

[B11] Chafekar S. M. Hoozemans J. J. M. Zwart R. Baas F. Scheper W. (2007). Aβ1–42induces mild endoplasmic reticulum stress in an aggregation State–dependent manner. Antioxid. Redox Signal. 9, 2245–2254. 10.1089/ars.2007.1797 17979527

[B12] Chang R. Suen K.-C. Ma C.-H. Elyaman W. Ng H.-K. Hugon J. (2002a). Involvement of double-stranded RNA-dependent protein kinase and phosphorylation of eukaryotic initiation factor-2alpha in neuronal degeneration. J. Neurochem. 83, 1215–1225. 10.1046/j.1471-4159.2002.01237.x 12437593

[B13] Chang R. C. Wong A. K. Ng H. K. Hugon J. (2002b). Phosphorylation of eukaryotic initiation factor 2 alpha (eIF2alpha) is associated with neuronal degeneration in Alzheimer’s disease. Neuroreport 13, 2429–2432. 10.1097/00001756-200212200-00011 12499843

[B14] Chen Z. Zhong C. (2013). Decoding Alzheimer’s disease from perturbed cerebral glucose metabolism: implications for diagnostic and therapeutic strategies. Prog. Neurobiol. 108, 21–43. 10.1016/j.pneurobio.2013.06.004 23850509

[B15] Colla E. Coune P. Liu Y. Pletnikova O. Troncoso J. C. Iwatsubo T.. (2012). Endoplasmic reticulum stress is important for the manifestations of α-synucleinopathy *in vivo*. J. Neurosci. 32, 3306–3320. 10.1523/jneurosci.5367-11.2012 22399753 PMC3461828

[B16] Combs C. K. (2009). Inflammation and microglia actions in Alzheimer’s disease. J. Neuroimmune Pharmacol. 4, 380–388. 10.1007/s11481-009-9165-3 19669893

[B17] Costa-Mattioli M. Gobert D. Harding H. Herdy B. Azzi M. Bruno M.. (2005). Translational control of hippocampal synaptic plasticity and memory by the eIF2alpha kinase GCN2. Nature 436, 1166–1173. 10.1038/nature03897 16121183 PMC1464117

[B18] Costa-Mattioli M. Gobert D. Stern E. Gamache K. Colina R. Cuello C.. (2007). eIF2α phosphorylation bidirectionally regulates the switch from short- to long-term synaptic plasticity and memory. Cell 129, 195–206. 10.1016/j.cell.2007.01.050 17418795 PMC4149214

[B19] Craft S. (2012). Alzheimer disease: insulin resistance and AD—extending the translational path. Nat. Rev. Neurol. 8, 360–362. 10.1038/nrneurol.2012.112 22710630

[B20] Crane P. K. Walker R. Hubbard R. A. Li G. Nathan D. M. Zheng H.. (2013). Glucose levels and risk of dementia. N. Engl. J. Med. 369, 540–548. 10.1056/NEJMoa1215740 23924004 PMC3955123

[B21] De Felice F. G. (2013). Alzheimer’s disease and insulin resistance: translating basic science into clinical applications. J. Clin. Invest. 123, 531–539. 10.1172/JCI64595 23485579 PMC3561831

[B22] De Felice F. G. Ferreira S. T. (2014). Inflammation, defective insulin signaling and mitochondrial dysfunction as common molecular denominators connecting type 2 diabetes to Alzheimer disease. Diabetes 63, 2262–2272. 10.2337/db13-1954 24931033

[B23] De Felice F. G. Lourenco M. V. Ferreira S. T. (2014). How does brain insulin resistance develop in Alzheimer’s disease? Alzheimers Dement. 10, S26–S32. 10.1016/j.jalz.2013.12.004 24529521

[B24] De Felice F. G. Velasco P. T. Lambert M. P. Viola K. Fernandez S. J. Ferreira S. T.. (2007). Aβ oligomers induce neuronal oxidative stress through an N-methyl-D-aspartate receptor-dependent mechanism that is blocked by the Alzheimer drug memantine. J. Biol. Chem. 282, 11590–11601. 10.1074/jbc.m607483200 17308309

[B25] de la Monte S. (2009). Insulin resistance and Alzheimer’s disease. BMB Rep. 42, 475–481. 10.5483/BMBRep.2009.42.8.475 19712582 PMC4600067

[B26] Devi L. Ohno M. (2010). Phospho-eIF2alpha level is important for determining abilities of BACE1 reduction to rescue cholinergic neurodegeneration and memory defects in 5XFAD mice. PLoS One 5:e12974. 10.1371/journal.pone.0012974 20886088 PMC2944882

[B27] Devi L. Ohno M. (2014). PERK mediates eIF2alpha phosphorylation responsible for BACE1 elevation, CREB dysfunction and neurodegeneration in a mouse model of Alzheimer’s disease. Neurobiol. Aging 35, 2272–2281. 10.1016/j.neurobiolaging.2014.04.031 24889041 PMC4127890

[B28] Dhawan G. Floden A. M. Combs C. K. (2012). Amyloid-β oligomers stimulate microglia through a tyrosine kinase mechanism. Neurobiol. Aging 33, 2247–2261. 10.1016/j.neurobiolaging.2011.10.027 22133278 PMC3294077

[B29] Di Prisco G. V. Huang W. Buffington S. A. Hsu C. C. Bonnen P. E. Placzek A. N.. (2014). Translational control of mGluR-dependent long-term depression and object-place learning by eIF2alpha. Nat. Neurosci. 17, 1073–1082. 10.1038/nn.3754 24974795 PMC4340591

[B30] Ferreira S. T. Clarke J. R. Bomfim T. R. De Felice F. G. (2014). Inflammation, defective insulin signaling and neuronal dysfunction in Alzheimer’s disease. Alzheimers Dement. 10(1 Suppl), S76–S83. 10.1016/j.jalz.2013.12.010 24529528

[B31] Ferreira S. T. Klein W. L. (2011). The Aβ oligomer hypothesis for synapse failure and memory loss in Alzheimer’s disease. Neurobiol. Learn. Mem. 96, 529–543. 10.1016/j.nlm.2011.08.003 21914486 PMC4390395

[B32] Flexner L. B. Flexner J. B. Roberts R. B. De La Haba G. (1964). Loss of recent memory in mice as related to regional inhibition of cerebral protein synthesis. Proc. Natl. Acad. Sci. U S A 52, 1165–1169. 10.1073/pnas.52.5.1165 14231435 PMC300416

[B33] Floden A. M. Combs C. K. (2006). Beta-amyloid stimulates murine postnatal and adult microglia cultures in a unique manner. J. Neurosci. 26, 4644–4648. 10.1523/jneurosci.4822-05.2006 16641245 PMC6674057

[B34] Forny-Germano L. Lyra E Silva N. M. Batista A. F. Brito-Moreira J. Gralle M. Boehnke S. E.. (2014). Alzheimer’s disease-like pathology induced by amyloid-beta oligomers in nonhuman primates. J. Neurosci. 34, 13629–13643. 10.1523/JNEUROSCI.1353-14.2014 25297091 PMC6608380

[B35] Gong Y. Chang L. Viola K. L. Lacor P. N. Lambert M. P. Finch C. E.. (2003). Alzheimer’s disease-affected brain: presence of oligomeric Aβ ligands (ADDLs) suggests a molecular basis for reversible memory loss. Proc. Natl. Acad. Sci. U S A 100, 10417–10422. 10.1073/pnas.1834302100 12925731 PMC193576

[B36] Gregor M. F. Hotamisligil G. S. (2011). Inflammatory mechanisms in obesity. Annu. Rev. Immunol. 29, 415–445. 10.1146/annurev-immunol-031210-101322 21219177

[B37] Halliday M. Radford H. Sekine Y. Moreno J. Verity N. Le Quesne J.. (2015). Partial restoration of protein synthesis rates by the small molecule ISRIB prevents neurodegeneration without pancreatic toxicity. Cell Death Dis. 6:e1672. 10.1038/cddis.2015.49 25741597 PMC4385927

[B38] Heneka M. T. Golenbock D. T. Latz E. (2015). Innate immunity in Alzheimer’s disease. Nat. Immunol. 16, 229–236. 10.1038/ni.3102 25689443

[B39] Hensley K. Floyd R. A. Zheng N.-Y. Nael R. Robinson K. A. Nguyen X.. (1999). p38 kinase is activated in the Alzheimer’s disease brain. J. Neurochem. 72, 2053–2058. 10.1046/j.1471-4159.1999.0722053.x 10217284

[B40] Herrup K. (2010). Reimagining Alzheimer’s disease—an age-based hypothesis. J. Neurosci. 30, 16755–16762. 10.1523/JNEUROSCI.4521-10.2010 21159946 PMC3004746

[B41] Hetz C. (2012). The unfolded protein response: controlling cell fate decisions under ER stress and beyond. Nat. Rev. Mol. Cell Biol. 13, 89–102. 10.1038/nrm3270 22251901

[B42] Hetz C. Chevet E. Harding H. P. (2013). Targeting the unfolded protein response in disease. Nat. Rev. Drug Discov. 12, 703–719. 10.1038/nrd3976 23989796

[B43] Hetz C. Mollereau B. (2014). Disturbance of endoplasmic reticulum proteostasis in neurodegenerative diseases. Nat. Rev. Neurosci. 15, 233–249. 10.1038/nrn3689 24619348

[B44] Hetz C. Thielen P. Matus S. Nassif M. Court F. Kiffin R.. (2009). XBP-1 deficiency in the nervous system protects against amyotrophic lateral sclerosis by increasing autophagy. Genes Dev. 23, 2294–2306. 10.1101/gad.1830709 19762508 PMC2758741

[B45] Hoozemans J. J. M. Van Haastert E. S. Nijholt D. A. T. Rozemuller A. J. M. Eikelenboom P. Scheper W. (2009). The unfolded protein response is activated in pretangle neurons in Alzheimer’s disease hippocampus. Am. J. Pathol. 174, 1241–1251. 10.2353/ajpath.2009.080814 19264902 PMC2671357

[B46] Hoozemans J. J. M. Veerhuis R. Haastert E. S. Rozemuller J. M. Baas F. Eikelenboom P.. (2005). The unfolded protein response is activated in Alzheimer’s disease. Acta Neuropathol. 110, 165–172. 10.1007/s00401-005-1038-0 15973543

[B47] Hotamisligil G. S. (2006). Inflammation and metabolic disorders. Nature 444, 860–867. 10.1038/nature05485 17167474

[B48] Hotamisligil G. S. Arner P. Caro J. F. Atkinson R. L. Spiegelman B. M. (1995). Increased adipose tissue expression of tumor necrosis factor-a in human obesity and insulin resistance. J. Clin. Invest. 95, 2409–2415. 10.1172/jci117936 7738205 PMC295872

[B49] Hotamisligil G. S. Peraldi P. Budavari A. Ellis R. White M. F. Spiegelman B. M. (1996). IRS-1-mediated inhibition of insulin receptor tyrosine kinase activity in TNF-α- and obesity-induced insulin resistance. Science 271, 665–668. 10.1126/science.271.5249.665 8571133

[B50] Hoyer S. Oesterreich K. Wagner O. (1988). Glucose metabolism as the site of the primary abnormality in early-onset dementia of Alzheimer type? J. Neurol. 235, 143–148. 10.1007/bf00314304 3367161

[B51] Kalmijin S. Launer L. J. Ott A. Witteman J. C. Hofman A. Breteler M. M. (1997). Dietary fat intake and the risk of incident dementia in the Rotterdam study. Ann. Neurol. 42, 776–782. 10.1002/ana.410420514 9392577

[B52] Kapogiannis D. Mattson M. P. (2011). Disrupted energy metabolism and neuronal circuit dysfunction in cognitive impairment and Alzheimer’s disease. Lancet Neurol. 10, 187–198. 10.1016/S1474-4422(10)70277-5 21147038 PMC3026092

[B53] Kim H.-J. Raphael A. R. Ladow E. S. Mcgurk L. Weber R. A. Trojanowski J. Q.. (2013). Therapeutic modulation of eIF2α phosphorylation rescues TDP-43 toxicity in amyotrophic lateral sclerosis disease models. Nat. Genet. 46, 152–160. 10.1038/ng.2853 24336168 PMC3934366

[B54] Kiyota T. Okuyama S. Swan R. J. Jacobsen M. T. Gendelman H. E. Ikezu T. (2010). CNS expression of anti-inflammatory cytokine interleukin-4 attenuates Alzheimer’s disease-like pathogenesis in APP+PS1 bigenic mice. FASEB J. 24, 3093–3102. 10.1096/fj.10-155317 20371618 PMC2909296

[B55] Kleinridders A. Cai W. Cappellucci L. Ghazarian A. Collins W. R. Vienberg S. G.. (2015). Insulin resistance in brain alters dopamine turnover and causes behavioral disorders. Proc. Natl. Acad. Sci. U S A 112, 3463–3468. 10.1073/pnas.1500877112 25733901 PMC4371978

[B56] Lai E. Teodoro T. Volchuk A. (2007). Endoplasmic reticulum stress: signaling the unfolded protein response. Physiology (Bethesda) 22, 193–201. 10.1152/physiol.00050.2006 17557940

[B57] Lajole P. Snapp E. L. (2011). Changes in BiP availability reveal hypersensitivity to acute endoplasmic reticulum stress in cells expressing mutant huntingtin. J. Cell Sci. 124, 3332–3343. 10.1242/jcs.087510 21896647 PMC3178454

[B58] Lambert M. P. Barlow A. K. Chromy B. A. Edwards C. Freed R. Liosatos M.. (1998). Diffusible, nonfibrillar ligands derived from Abeta1–42 are potent central nervous system neurotoxins. Proc. Natl. Acad. Sci. U S A 95, 6448–6453. 10.1073/pnas.95.11.6448 9600986 PMC27787

[B59] Lange P. S. Chavez J. C. Pinto J. T. Coppola G. Sun C. W. Townes T. M.. (2008). ATF4 is an oxidative stress-inducible, prodeath transcription factor in neurons *in vitro* and *in vivo*. J. Exp. Med. 205, 1227–1242. 10.1084/jem.20071460 18458112 PMC2373852

[B60] Ledo J. H. Azevedo E. P. Clarke J. R. Ribeiro F. C. Figueiredo C. P. Foguel D.. (2013). Amyloid-β oligomers link depressive-like behavior and cognitive deficits in mice. Mol. Psychiatry 18, 1053–1054. 10.1038/mp.2012.168 23183490 PMC3781315

[B61] Lee D. Y. Lee K.-S. Lee H. J. Kim D. H. Noh Y. H. Yu K.. (2010). Activation of PERK signaling attenuates Abeta-mediated ER stress. PloS One 5:e10489. 10.1371/journal.pone.0010489 20463975 PMC2864758

[B62] Lourenco M. V. Clarke J. R. Frozza R. L. Bomfim T. R. Forny-Germano L. Batista A. F.. (2013). TNF-α mediates PKR-dependent memory impairment and brain IRS-1 inhibition induced by Alzheimer’s β-amyloid oligomers in mice and monkeys. Cell Metab. 18, 831–843. 10.1016/j.cmet.2013.11.002 24315369

[B63] Lourenco M. V. Ledo J. H. (2013). Targeting Alzheimer’s pathology through PPARγ signaling: modulation of microglial function. J. Neurosci. 33, 5083–5084. 10.1523/JNEUROSCI.0172-13.2013 23516274 PMC6705006

[B64] Ma T. Trinh M. A. Wexler A. J. Bourbon C. Gatti E. Pierre P.. (2013). Suppression of eIF2α kinases alleviates Alzheimer’s disease–related plasticity and memory deficits. Nat. Neurosci. 16, 1299–1305. 10.1038/nn.3486 23933749 PMC3756900

[B65] Ma Q. L. Yang F. Rosario E. R. Ubeda O. J. Beech W. Gant D. J.. (2009). β-amyloid oligomers induce phosphorylation of tau and inactivation of insulin receptor substrate via c-Jun N-terminal kinase signaling: suppression by omega-3 fatty acids and curcumin. J. Neurosci. 29, 9078–9089. 10.1523/JNEUROSCI.1071-09.2009 19605645 PMC3849615

[B66] Masters C. L. Simms G. Weinman N. A. Multhaup G. Mcdonald B. L. Beyreuther K. (1985). Amyloid plaque core protein in Alzheimer disease and down syndrome. Proc. Natl. Acad. Sci. U S A 82, 4245–4249. 10.1073/pnas.82.12.4245 3159021 PMC397973

[B67] Matsuzaki T. Sasaki K. Tanizaki Y. Hata J. Fujimi K. Matsui Y.. (2010). Insulin resistance is associated with the pathology of Alzheimer disease: the Hisayama Study. Neurology 75, 764–770. 10.1212/wnl.0b013e3181eee25f 20739649

[B68] Mattson M. P. (2008). Hormesis defined. Ageing Res. Rev. 7, 1–7. 10.1016/j.arr.2007.08.007 18162444 PMC2248601

[B69] Mattson M. P. (2012). Energy intake and exercise as determinants of brain health and vulnerability to injury and disease. Cell Metab. 16, 706–722. 10.1016/j.cmet.2012.08.012 23168220 PMC3518570

[B70] McAlpine F. E. Lee J. Harms A. S. Ruhn K. A. Blurton-Jones M. Hong J.. (2009). Inhibition of soluble TNF signaling in a mouse model of Alzheimer’s disease prevents pre-plaque amyloid-associated neuropathology. Neurobiol. Dis. 34, 163–177. 10.1016/j.nbd.2009.01.006 19320056 PMC2948857

[B71] Medeiros R. Prediger R. D. S. Passos G. F. Pandolfo P. Duarte F. S. Franco J. L.. (2007). Connecting TNF-α signaling pathways to iNOS expression in a mouse model of Alzheimer’s disease: relevance for the behavioral and synaptic deficits induced by amyloid-β protein. J. Neurosci. 27, 5394–5404. 10.1523/jneurosci.5047-06.2007 17507561 PMC6672347

[B72] Medinas D. B. Hetz C. (2013). Proteostasis impairment: at the intersection between Alzheimer’s disease and diabetes. Cell Metab. 18, 771–772. 10.1016/j.cmet.2013.11.009 24315366

[B73] Mitsuda T. Hayakawa Y. Itoh M. Ohta K. Nakagawa T. (2007). ATF4 regulates gamma-secretase activity during amino acid imbalance. Biochem. Biophys. Res. Commun. 352, 722–727. 10.1016/j.bbrc.2006.11.075 17141186

[B74] Moloney A. M. Griffin R. J. Timmons S. O’connor R. Ravid R. O’neill C. (2010). Defects in IGF-1 receptor, insulin receptor and IRS-1/2 in Alzheimer’s disease indicate possible resistance to IGF-1 and insulin signalling. Neurobiol. Aging 31, 224–243. 10.1016/j.neurobiolaging.2008.04.002 18479783

[B75] Monson N. L. Ireland S. J. Ligocki A. J. Chen D. Rounds W. H. Li M.. (2014). Elevated CNS inflammation in patients with preclinical Alzheimer’s disease. J. Cereb. Blood Flow Metab. 34, 30–33. 10.1038/jcbfm.2013.183 24149932 PMC3887357

[B76] Moreno J. A. Halliday M. Molloy C. Radford H. Verity N. Axten J. M.. (2013). Oral treatment targeting the unfolded protein response prevents neurodegeneration and clinical disease in prion-infected mice. Sci. Transl. Med. 5:206ra138. 10.1126/scitranslmed.3006767 24107777

[B77] Moreno J. A. Radford H. Peretti D. Steinert J. R. Verity N. Martin M. G.. (2012). Sustained translational repression by eIF2α-P mediates prion neurodegeneration. Nature 485, 507–511. 10.1038/nature11058 22622579 PMC3378208

[B78] Morris G. P. Clark I. A. Vissel B. (2014). Inconsistencies and controversies surrounding the amyloid hypothesis of Alzheimer’s disease. Acta Neuropathol. Commun. 2:135. 10.1186/s40478-014-0135-5 25231068 PMC4207354

[B79] Mouton-Liger F. Paquet C. Dumurgier J. Bouras C. Pradier L. Gray F. (2012a). Oxidative stress increases BACE1 protein levels through activation of the PKR-eIF2α pathway. Biochimica et Biophysica Acta (BBA) - Mol. Basis Dis. 1822, 885–896. 10.1016/j.bbadis.2012.01.009 22306812

[B80] Mouton-Liger F. Paquet C. Dumurgier J. Lapalus P. Gray F. Laplanche J.-L.. (2012b). Increased cerebrospinal fluid levels of double-stranded RNA-dependent protein kinase in Alzheimer’s disease. Biol. Psychiatry 71, 829–835. 10.1016/j.biopsych.2011.11.031 22281122

[B81] Mucke L. Selkoe D. J. (2012). Neurotoxicity of amyloid-beta protein: synaptic and network dysfunction. Cold Spring Harb. Perspect. Med. 2:a006338. 10.1101/cshperspect.a006338 22762015 PMC3385944

[B82] Nakamura T. Kunz R. C. Zhang C. Kimura T. Yuan C. L. Baccaro B.. (2015). A critical role for PKR complexes with TRBP in immunometabolic regulation and eIF2alpha phosphorylation in obesity. Cell Rep. 11, 295–307. 10.1016/j.celrep.2015.03.021 25843719 PMC4439210

[B83] Nisticò R. Cavallucci V. Piccinin S. Macrì S. Pignatelli M. Mehdawy B.. (2012). Insulin receptor β-subunit haploinsufficiency impairs hippocampal late-phase LTP and recognition memory. Neuromolecular Med. 14, 262–269. 10.1007/s12017-012-8184-z 22661254

[B84] O’Connor T. Sadleir K. R. Maus E. Velliquette R. A. Zhao J. Cole S. L.. (2008). Phosphorylation of the translation initiation factor eIF2α increases BACE1 levels and promotes amyloidogenesis. Neuron 60, 988–1009. 10.1016/j.neuron.2008.10.047 19109907 PMC2667382

[B85] O’Neill C. (2013). PI3-kinase/Akt/mTOR signaling: impaired on/off switches in aging, cognitive decline and Alzheimer’s disease. Exp. Gerontol. 48, 647–653. 10.1016/j.exger.2013.02.025 23470275

[B86] Ott A. Stolk R. P. Hofman A. Van Harskamp F. Grobbee D. E. Breteler M. M. B. (1996). Association of diabetes mellitus and dementia: the rotterdam study. Diabetologia 39, 1392–1397. 10.1007/s001250050588 8933010

[B87] Ott A. Stolk R. P. Van Harskamp F. Pols H. A. P. Hofman A. Breteler M. M. B. (1999). Diabetes mellitus and the risk of dementia: the rotterdam study. Neurology 53, 1937–1937. 10.1212/wnl.53.9.1937 10599761

[B88] Ounallah-Saad H. Sharma V. Edry E. Rosenblum K. (2014). Genetic or pharmacological reduction of perk enhances cortical-dependent taste learning. J. Neurosci. 34, 14624–14632. 10.1523/jneurosci.2117-14.2014 25355215 PMC6608429

[B89] Ozcan U. Cao Q. Yilmaz E. Lee A. H. Iwakoshi N. N. Ozdelen E.. (2004). Endoplasmic reticulum stress links obesity, insulin action and type 2 diabetes. Science 306, 457–461. 10.1126/science.1103160 15486293

[B90] Ozcan U. Yilmaz E. Ozcan L. Furuhashi M. Vaillancourt E. Smith R. O.. (2006). Chemical chaperones reduce ER stress and restore glucose homeostasis in a mouse model of type 2 diabetes. Science 313, 1137–1140. 10.1126/science.1128294 16931765 PMC4741373

[B91] Pan X. D. Zhu Y. G. Lin N. Zhang J. Ye Q. Y. Huang H. P.. (2011). Microglial phagocytosis induced by fibrillar beta-amyloid is attenuated by oligomeric beta-amyloid: implications for Alzheimer’s disease. Mol. Neurodegener. 6:45. 10.1186/1750-1326-6-45 21718498 PMC3149591

[B92] Paquet C. Mouton-Liger F. Meurs E. F. Mazot P. Bouras C. Pradier L.. (2011). The PKR activator PACT is induced by Aβ: involvement in Alzheimer’s disease. Brain Pathol. 22, 219–229. 10.1111/j.1750-3639.2011.00520.x 21790829 PMC8029131

[B93] Parajuli B. Sonobe Y. Horiuchi H. Takeuchi H. Mizuno T. Suzumura A. (2013). Oligomeric amyloid beta induces IL-1beta processing via production of ROS: implication in Alzheimer’s disease. Cell Death Dis. 4:e975. 10.1038/cddis.2013.503 24357806 PMC3877570

[B94] Prince M. Bryce R. Albanese E. Wimo A. Ribeiro W. Ferri C. P. (2013). The global prevalence of dementia: a systematic review and metaanalysis. Alzheimers Dement. 9, 63.e2–75.e2. 10.1016/j.jalz.2012.11.007 23305823

[B95] Rajasethupathy P. Antonov I. Sheridan R. Frey S. Sander C. Tuschl T.. (2012). A role for neuronal pirnas in the epigenetic control of memory-related synaptic plasticity. Cell 149, 693–707. 10.1016/j.cell.2012.02.057 22541438 PMC3442366

[B96] Raven J. F. Koromilas A. E. (2008). PERK and PKR: old kinases learn new tricks. Cell Cycle 7, 1146–1150. 10.4161/cc.7.9.5811 18418049

[B97] Ricobaraza A. Cuadrado-Tejedor M. Marco S. Pérez-Otaño I. García-Osta A. (2010). Phenylbutyrate rescues dendritic spine loss associated with memory deficits in a mouse model of Alzheimer disease. Hippocampus 22, 1040–1050. 10.1002/hipo.20883 21069780

[B98] Ricobaraza A. Cuadrado-Tejedor M. Perez-Mediavilla A. Frechilla D. Del Rio J. Garcia-Osta A. (2009). Phenylbutyrate ameliorates cognitive deficit and reduces tau pathology in an Alzheimer’s disease mouse model. Neuropsychopharmacology 34, 1721–1732. 10.1038/npp.2008.229 19145227

[B99] Roffé M. Hajj G. N. Azevedo H. F. Alves V. S. Castilho B. A. (2013). IMPACT is a developmentally regulated protein in neurons that opposes the eukaryotic initiation factor 2alpha kinase GCN2 in the modulation of neurite outgrowth. J. Biol. Chem. 288, 10860–10869. 10.1074/jbc.m113.461970 23447528 PMC3624466

[B100] Rossato J. I. Bevilaqua L. R. Myskiw J. C. Medina J. H. Izquierdo I. Cammarota M. (2007). On the role of hippocampal protein synthesis in the consolidation and reconsolidation of object recognition memory. Learn. Mem. 14, 36–46. 10.1101/lm.422607 17272651 PMC1838544

[B101] Schubert M. Gautam D. Surjo D. Ueki K. Baudler S. Schubert D.. (2004). Role for neuronal insulin resistance in neurodegenerative diseases. Proc. Natl. Acad. Sci. U S A 101, 3100–3105. 10.1073/pnas.0308724101 14981233 PMC365750

[B102] Segev Y. Michaelson D. M. Rosenblum K. (2012). ApoE ε4 is associated with eIF2α phosphorylation and impaired learning in young mice. Neurobiol. Aging 34, 863–872. 10.1016/j.neurobiolaging.2012.06.020 22883908

[B103] Selkoe D. J. (2002). Alzheimer’s disease is a synaptic failure. Science 298, 789–791. 10.1126/science.1074069 12399581

[B104] Sondag C. M. Dhawan G. Combs C. K. (2009). Beta amyloid oligomers and fibrils stimulate differential activation of primary microglia. J. Neuroinflammation 6:1. 10.1186/1742-2094-6-1 19123954 PMC2632990

[B105] Steen E. Terry B. M. Rivera E. J. Cannon J. L. Neely T. R. Tavares R.. (2005). Impaired insulin and insulin-like growth factor expression and signaling mechanisms in Alzheimer’s disease – is this type 3 diabetes? J. Alzheimers Dis. 7, 63–80. 15750215 10.3233/jad-2005-7107

[B106] Stern E. Chinnakkaruppan A. David O. Sonenberg N. Rosenblum K. (2013). Blocking the eIF2α kinase (PKR) enhances positive and negative forms of cortex-dependent taste memory. J. Neurosci. 33, 2517–2525. 10.1523/JNEUROSCI.2322-12.2013 23392680 PMC6619168

[B107] Talbot K. Wang H. Kazi H. Han L. Bakshi K. P. Stucky A.. (2012). Demonstrated brain insulin resistance in Alzheimer’s disease patients is associated with IGF-1 resistance, IRS-1 dysregulation and cognitive decline. J. Clin. Invest. 122, 1316–1338. 10.1172/JCI59903 22476197 PMC3314463

[B108] Vallerie S. N. Hotamisligil G. S. (2010). The role of JNK proteins in metabolism. Sci. Transl. Med. 2:60rv65. 10.1126/scitranslmed.3001007 21123811

[B109] van der Harg J. M. Nölle A. Zwart R. Boerema A. S. Van Haastert E. S. Strijkstra A. M.. (2014). The unfolded protein response mediates reversible tau phosphorylation induced by metabolic stress. Cell Death Dis. 5:e1393. 10.1038/cddis.2014.354 25165879 PMC4454326

[B110] Viola K. L. Klein W. L. (2015). Amyloid beta oligomers in Alzheimer’s disease pathogenesis, treatment and diagnosis. Acta Neuropathol. 129, 183–206. 10.1016/j.bpj.2014.11.1128 25604547 PMC4390393

[B111] Wang R. Li J. J. Diao S. Kwak Y. D. Liu L. Zhi L.. (2013). Metabolic stress modulates Alzheimer’s beta-secretase gene transcription via SIRT1-PPARgamma-PGC-1 in neurons. Cell Metab. 17, 685–694. 10.1016/j.cmet.2013.03.016 23663737 PMC5396538

[B112] Wiley J. C. Pettan-Brewer C. Ladiges W. C. (2011). Phenylbutyric acid reduces amyloid plaques and rescues cognitive behavior in AD transgenic mice. Aging Cell 10, 418–428. 10.1111/j.1474-9726.2011.00680.x 21272191

[B113] Xia W. Yang T. Shankar G. M. Smith I. M. Shen Y. Walsh D.. (2009). A specific enzyme-linked immunosorbent assay for measuring β-amyloid protein oligomers in human plasma and brain tissue of patients with Alzheimer disease. Arch. Neurol. 66, 190–199. 10.1001/archneurol.2008.565 19204155 PMC3618974

[B114] Yamanaka M. Ishikawa T. Griep A. Axt D. Kummer M. P. Heneka M. T. (2012). PPARγ/RXRα-induced and CD36-mediated microglial amyloid-β phagocytosis results in cognitive improvement in amyloid precursor protein/presenilin 1 mice. J. Neurosci. 32, 17321–17331. 10.1523/JNEUROSCI.1569-12.2012 23197723 PMC6621845

[B115] Yang Y. M. Shang D. S. Zhao W. D. Fang W. G. Chen Y. H. (2013). Microglial TNF-alpha-dependent elevation of MHC class I expression on brain endothelium induced by amyloid-beta promotes T cell transendothelial migration. Neurochem. Res. 38, 2295–2304. 10.1007/s11064-013-1138-5 23990225

[B116] Yoon S. O. Park D. J. Ryu J. C. Ozer H. G. Tep C. Shin Y. J.. (2012). JNK3 perpetuates metabolic stress induced by Aβ peptides. Neuron 75, 824–837. 10.3410/f.717965605.793466623 22958823 PMC3438522

[B117] Zhu P. J. Huang W. Kalikulov D. Yoo J. W. Placzek A. N. Stoica L.. (2011). Suppression of PKR promotes network excitability and enhanced cognition by interferon-γ-mediated disinhibition. Cell 147, 1384–1396. 10.1016/j.cell.2011.11.029 22153080 PMC3569515

